# Manganese(II) and Zinc(II) metal complexes of novel bidentate formamide-based Schiff base ligand: synthesis, structural characterization, antioxidant, antibacterial, and *in-silico* molecular docking study

**DOI:** 10.3389/fchem.2024.1414646

**Published:** 2024-07-19

**Authors:** Vijay Kumar Juyal, Shweta Chand Thakuri, Mohit Panwar, Rashmi, Om Prakash, Kahkashan Perveen, Najat A. Bukhari, Viveka Nand

**Affiliations:** ^1^ Department of Chemistry, G.B. Pant University of Agriculture and Technology, Pantnagar, India; ^2^ Regional Ayurveda Research Institute, Ministry of Ayush, Gwalior, India; ^3^ Department of Botany and Microbiology, College of Science, King Saud University Riyadh, Riyadh, Saudi Arabia

**Keywords:** formamide, Schiff base, antibacterial, antioxidant, *in silico* molecular docking

## Abstract

A new bidentate Schiff base ligand (C_16_H_16_Cl_2_N_4_), condensation product of ethylene diamine and 4-chloro N-phenyl formamide, and its metal complexes [M(C_16_H_16_Cl_2_N_4_)_2_(OAc)_2_] (where M = Mn(II) and Zn(II)) were synthesized and characterized using various analytical and spectral techniques, including high-resolution mass spectrometry (HRMS), elemental analysis, ultraviolet–visible (UV–vis), Fourier-transform infrared (FTIR) spectroscopy, AAS, molar conductance, ^1^H NMR, and powder XRD. All the compounds were non-electrolytes and nanocrystalline. The synthesized compounds were assessed for antioxidant potential by DPPH radical scavenging and FRAP assay, with BHT serving as the positive control. Inhibitory concentration at 50% inhibition (IC_50_) values were calculated and used for comparative analysis. Furthermore, the prepared compounds were screened for antibacterial activity against two Gram-negative bacteria (*Staphylococcus aureus* and *Bacillus subtilis*) and two Gram-positive bacteria (*Escherichia coli* and *Salmonella typhi*) using disk-diffusion methods, with amikacin employed as the standard reference. The comparison of inhibition zones revealed that the complexes showed better antibacterial activity than the ligand. To gain insights into the molecular interactions underlying the antibacterial activity, the ligand and complexes were analyzed for their binding affinity with *S. aureus* tyrosyl–tRNA synthetase (PDB ID: 1JIL) and *S. typhi* cell membrane protein OmpF complex (PDB ID: 4KR4). These analyses revealed robust interactions, validating the observed antibacterial effects against the tested bacterial strains.

## 1 Introduction

Schiff bases, also referred to as azomethines (>C=N-) or imines, are compounds synthesized through the reaction of primary amines with ketones or aldehydes under specific conditions (Kanwal et al., 2022). These compounds are extensively studied because of their sigma donor tendency toward metal and π acceptor properties in imine nitrogen atoms. This distinctive property renders it a valuable donating ligand in coordination chemistry (Bruns et al., 2010; Abd El-Lateef et al., 2018). Schiff base ligands containing nitrogen, along with other donor atoms such as oxygen and sulfur in their molecular structures, serve as chelating agents and easily form complexes with various metal ions (Abd El Wahed et al., 2004; Mishra et al., 2005; El-Sherif and Eldebss, 2011). In recent years, these complexes gained significant attention due to their diverse use in biology (Ghanghas et al., 2021), as models for metal-containing sites in metalloproteins (Ueno et al., 2004; Shahraki, 2022), catalysts for some organic reactions (Juyal et al., 2023a), and complexing ability toward some toxic metals (Vaghasiya et al., 2004) encompassing antibacterial (Kargar et al., 2021; Muthukumar et al., 2022), antifungal (Joshi et al., 2020; Shiryaev et al., 2021; Borrego-Muñoz et al., 2022), anticancer (Tadele and Tsega, 2019; Aslan et al., 2020; Alorini et al., 2022), antioxidant (Buldurun et al., 2019; Bingöl and Turan, 2020; Yadav et al., 2021), anti-inflammatory (Devi et al., 2019; Krishna et al., 2023), and antiviral activities (Abd El-Hamid et al., 2023; Bhandarkar et al., 2023).

The formylation-driven amination process, typically achieved by combining amines with formic acids, results in the formation of formamide. These compounds hold significant importance in both organic and industrial chemistry (Liang et al., 2023). N-Alkylformamides, polar solvents, like diethylformamide and dimethylformamide, are extensively utilized in both chemical laboratories and industrial settings for the synthesis of films, artificial fibers, and leather products (Amato et al., 2001). Formamides exhibit dual reactivity as both electrophilic and nucleophilic agents (Muzart, 2009), serving as a versatile source of key intermediates that mediate various reactions. Their structural flexibility allows engagement in diverse reactions functioning as building blocks for diverse units like -CHO, -O, -CONMe_2_, -NMe_2_, - CO, and -Me (Ding and Jiao, 2012). Formamides serve as intermediates in the synthesis of pharmaceutically active molecules including fluoroquinolones like 1,2-dihydroquinolines (Kobayashi et al., 1995), norfloxacin, and ciprofloxacin (Jackson and Meth-Cohn, 1995) and nitrogen-bridged heterocycles such as oxazolidinones (Lohray et al., 1999) and benzimidazole (Mohanty et al., 2018). However, metal complexes of formamide-based moieties are not widely studied, so there is untapped potential for discovery and advancement in the understanding of metal complexes with formamide-based moieties.

The overproduction of reactive oxygen species, like OH^**.**^ and superoxide anions, is recognized for inducing oxidative harm to DNA, lipids, and proteins. This contributes significantly to cancer, aging, inflammation, cardiovascular diseases, and neurodegenerative disorders (Beyazit et al., 2020). To counteract these harmful effects, antioxidants are crucial. Various studies have indicated the potential of Schiff base metal complexes as efficient scavengers of these free radicals (Sharma et al., 2015; Mumtaz et al., 2020). Furthermore, pathogenic microorganisms pose a substantial threat through life-threatening bacterial infections (Xu et al., 2020). Although antibiotics and antibacterial agents have been the traditional means to combat harmful bacterial growth, their prolonged use has raised concerns about bacterial resistance (Kaya et al., 2021). Consequently, there is an ongoing demand for novel antibacterial agents. Antioxidants can modulate the redox balance within microbial cells by scavenging free radicals and maintaining cellular redox homeostasis. Disruption of the redox balance can interfere with essential metabolic processes in microbes, leading to growth inhibition or cell death (Zandi and Schnug, 2022). In the given context, metal complexes of Schiff bases have emerged as promising candidates (Chen et al., 2017; El-Gammal et al., 2021).

Molecular docking techniques are instrumental in drug design and mechanistic studies, enabling the precise positioning of molecules within the binding sites of target macromolecules without forming chemical bonds (Juyal et al., 2023b). The process of docking utilizes computer simulations to predict how small compounds or macromolecules will interact with receptors at a molecular level, utilizing specialized docking software such as DOCK (Ismael et al., 2018) and AutoDock (Cheng et al., 2009; Junaid et al., 2018). These techniques offer accurate and efficient predictions of favorable interactions between proteins and ligands. These programs function as powerful computational filters, streamlining the search for potential bioactive compounds before experimental screening, thus reducing both costs and labor. Additionally, they contribute significantly to the understanding of molecular mechanisms post-experimental screening (Pagadala et al., 2017).

The present study aims to create potent antioxidant and antibacterial compounds with metal complexes of formamide-based Schiff base moieties due to their unique structural framework. The inclusion of the imine group enhances biological activity through hydrogen bonding, thus motivating the synthesis of the targeted molecules. The ligand precursor formamide was synthesized through chloroanilines and formic acid, utilizing NH_2_OH.HCl as the catalyst. The ligand was then synthesized by a reaction of the ligand precursor with ethylene diamine.

Manganese (Mn) and zinc (Zn) are essential trace elements crucial for numerous biological functions. Mn supports the activities of enzymes like arginase and manganese superoxide dismutase, aiding the urea cycle and oxidative stress protection (Bowman et al., 2011). Zn is integral to over 300 enzymes, including DNA polymerase, and is vital for DNA replication and repair, protein integrity, and immune signaling (Chasapis et al., 2020). Their unique properties of Mn(II) and Zn(II) make them indispensable in cellular roles and drives the synthesis of complexes. The incorporation of Mn(II) and Zn(II) into the ligand was achieved by chelation of the synthesized ligand to different metal salts at a 2:1 M ratio. The synthesized compounds were subjected to biological assessments, including assessment of antioxidant and antibacterial activities, to evaluate the responsiveness of compounds to various microorganisms. Additionally, a molecular docking study was conducted to gain insights into the bioactive mechanisms of these compounds.

## 2 Materials and methods

### 2.1 Reagents

The reagents Mn(OAc)_2_.4H_2_O (Sigma-Aldrich), Zn(OAc)_2_.2H_2_O (Sigma-Aldrich), ethylene diamine (Sigma-Aldrich), formic acid (HiMedia), methanol (Molychem), 4-chloroaniline (Molychem), ethanol (Molychem), and hydroxylamine hydrochloride (Sigma-Aldrich) were utilized in their highest degree of purity (AR grade). The bacterial culture was collected from the Department of Microbiology, CVAS, GB Pant University of Agriculture and Technology, Pantnagar.

### 2.2 Instruments

Molar conductivity of all compounds was determined using the Systronics conductivity TDS meter 308, while the melting point was determined using the Decibel DB-3135H MP apparatus. Elemental analysis was carried out using the vario MICRO cube (Elementar Analysensysteme, Germany). Moreover, the metal analysis was performed using the AAS instrument Element AS AAS 4141, and ^1^H NMR was carried out using the JEOL JNM ECS400 (400 MHz, dimethyl sulfoxide (DMSO)-d6). The magnetic molar susceptibilities (χ_M_) were gauged using Quincke’s tube in conjunction with the Digital Gauss Meter, DGM-102, and µeff was then calculated using the expression µeff = 2.828 (χ_M_.T)^1/2^ B.M. (Abou-Hussein and Linert, 2015). Powder XRD (PXRD) was conducted using the Bruker D8 ADVANCE diffractometer, while the Fourier-transform infrared (FTIR) spectroscopy data were collected by using the PerkinElmer FTIR spectrophotometer (350–4,000 cm^−1^). The ultraviolet–visible (UV–vis) spectra were measured (0.05 g/L) using the US GENESYS 10S spectrophotometer (Thermo Fisher Scientific).

### 2.3 Synthesis

#### 2.3.1 Preparation of the ligand

The preparation of the ligand involves two steps. First, the precursor formamide was synthesized and then ethylene diamine was attached to the formamide structure to attain the Schiff base ligand moiety.

##### 2.3.1.1 Synthesis of ligand precursors 4-chloro-(N-phenyl)formamide

**SCHEME 1:**
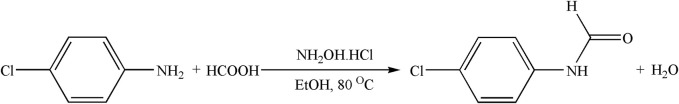
Synthesis of 4-chloro-(N-phenyl)formamide (Schiff base ligand precursors).

Formic acid (2 mmol) and 4-chloroaniline (1 mmol) were refluxed together at 80°C with the catalyst NH_2_OH.HCl (Scheme 1). The reaction progress was monitored through thin-layer chromatography (TLC.), and once the reaction was concluded, EtOAc was poured into the mixture. Subsequently, the reaction mixture was washed with H_2_O and 5% HCl solution (three times). The mixture was then dried with anhydrous Na_2_SO_4_, and the solvent was removed by vacuum distillation. A white powder product was obtained with good yield.

**Ligand precursor [C**_**7**_ **H**_**6**_ **ClNO]:** White; yield: 84%; MP 53°C; ^1^H NMR (400 MHz, DMSO-d6, TMS) δ (ppm): 3.8 (s, 1H, NH), 7.1–7.6 (m, 4H, C_6_H_6_), and 7.8 (s, 1H, CHO); and FTIR (KBr, cm^−1^): 3,515 (N-H), 3,052 (-CH), and 1,715 (C=O).

##### 2.3.1.2 Synthesis of the Schiff base ligand

**SCHEME 2:**
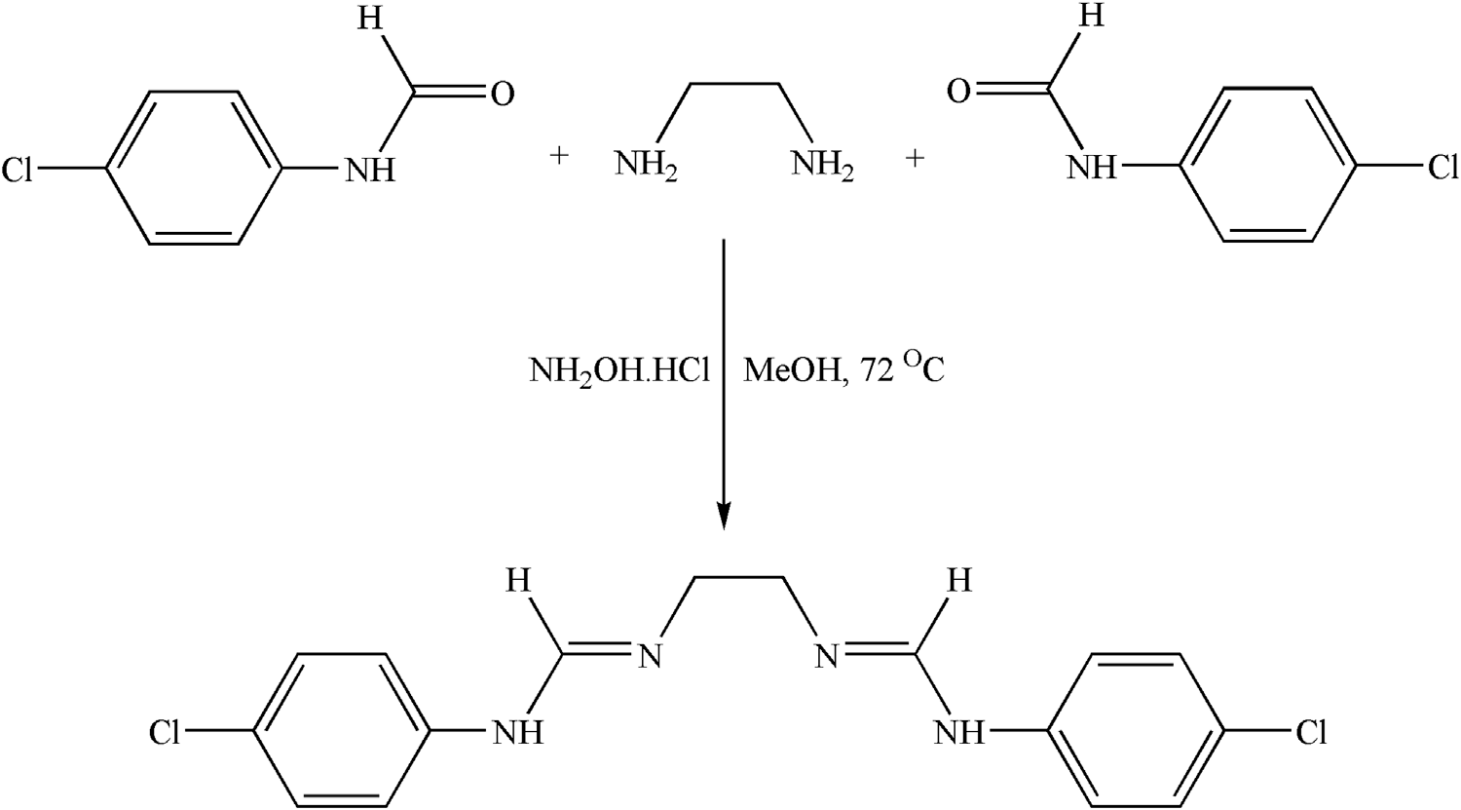
Synthetic route diagram of the Schiff base ligand.

A methanolic solution of ethylene diamine (1 mmol) was added dropwise to a methanolic solution of 4-chloro-(N-phenyl)formamide (2 mmol) in the presence of NH_2_OH.HCl at 72°C (Scheme 2). Once the reaction was concluded, EtOAc was introduced to the reaction mixture. Subsequently, the reaction mixture underwent successive washes with H_2_O and a 5% HCl solution (three times). Subsequently, the mixture was dried using anhydrous Na_2_SO_4_, and the solvent was removed through vacuum distillation. A light-yellow crude product was obtained, which was dried, washed, and recrystallized with C_2_H_5_OH.

**Schiff base ligand [C**_**16**_ **H**_**16**_ **Cl**_**2**_ **N**_**4**_ **]:** Light yellow; yield: 84%; MP 94°C; ^1^H NMR (400 MHz, DMSO-d6, TMS) δ (ppm): 1.5 (t, 2H, CH_2_), 4.2 (s, 1H, NH), 6.6–7.5 (m, 4H, C_6_H_6_), and 7.8 (s, 1H, CH); FTIR (KBr, cm^−1^): 3,536 (N-H), 2,972 (C-H), and 1,621 (C=N).

#### 2.3.2 General methods for the preparation of metal complexes

**SCHEME 3:**
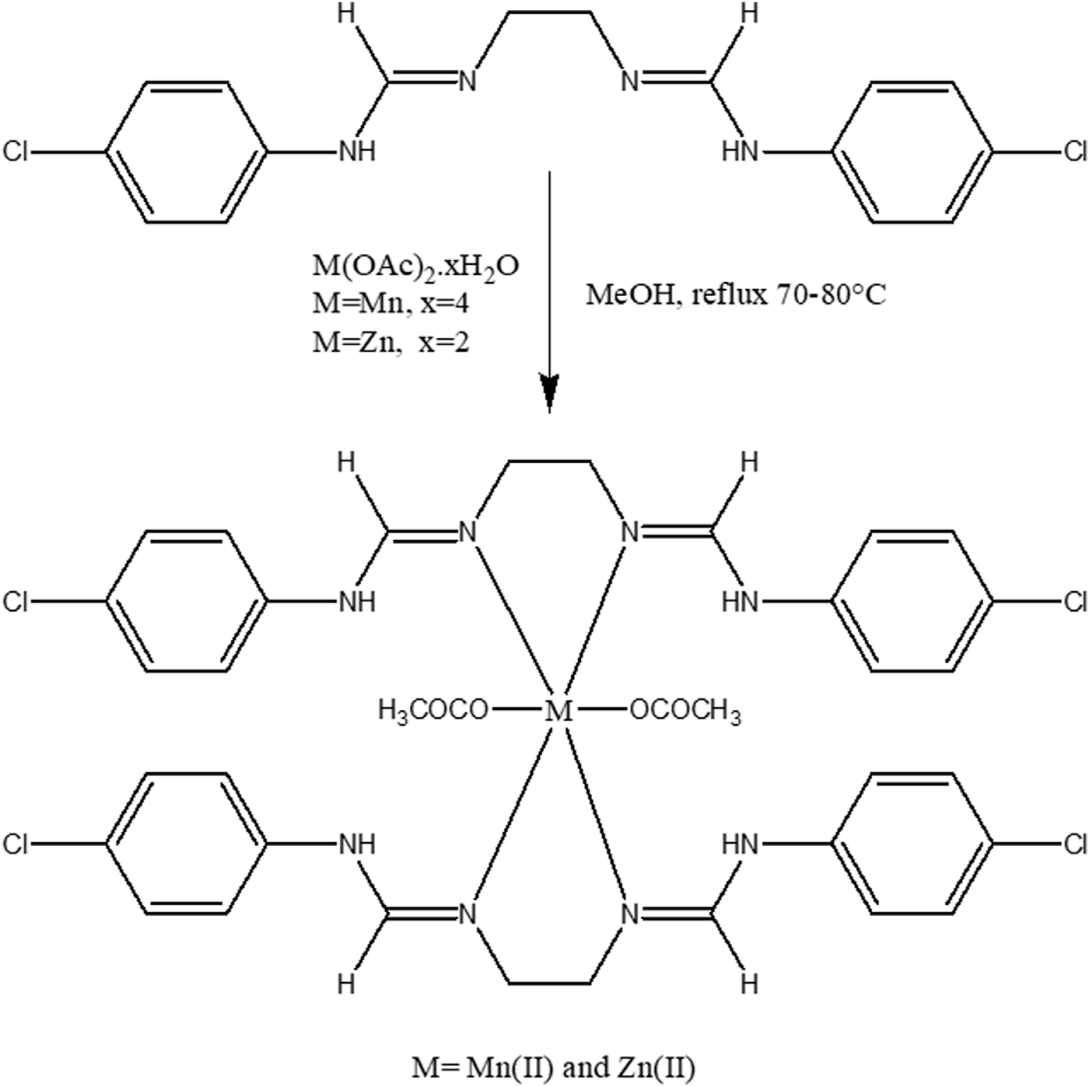
Synthesis of metal complexes of Mn(II) and Zn(II).

The Schiff base ligand (2 mmol methanolic solution) was added dropwise to the 1 mmol methanolic solution of metal salts (Mn(OAc)_2_.4H_2_O and Zn(OAc)_2_.2H_2_O). The resulting mixture was stirred for 8 h at a temperature range of 70°C–80°C (Scheme 3). Following this, the mixture was allowed to stand overnight, resulting in the precipitation of colored crystals with a yield of 72%–78%.

**Mn(II) complex [(C**_**16**_ **H**_**16**_ **Cl**_**2**_ **N**_**4**_ **)**_**2**_ **Mn(OAc)**_**2**_ **]:** Light pink; yield: 78%; decomposition temperature: 281°C; FTIR (KBr, cm^−1^): 3,547 (N-H), 2,971 (=CH), 1,713 (C=O), 1,675 (C=N), 1,521 (COO)_asymm._, 1,325 (COO)_symm._, 468 (Mn-N), and 378 (Mn-O).

**Zn(II) complex [(C**_**16**_ **H**_**16**_ **Cl**_**2**_ **N**_**4**_ **)**_**2**_ **Zn(OAc)**_**2**_ **]:** Yellow; yield: 75%; decomposition temperature: 277°C; ^1^H NMR (400 MHz, DMSO-d6, TMS) δ (ppm): 1.2 (*t*, 2H, CH2), 2.6 (s, 3H, COCH_3_), 3.4 (s, 2H, NH), 6.6–7.6 (m, 4H, C_6_H_6_), and 7.8 (s, 2H, CH); FTIR (KBr, cm^−1^): 3,568 (N-H), 2,972 (=C-H), 1,711 (C=O), 1,625 (C=N), 1,508 (COO)_asymm._, 1,334 (COO)_symm._, 456 (Zn-O), and 396 (Zn-N).

### 2.4 Biological activities

#### 2.4.1 Antioxidant activity

To assess the antioxidant potential of the compounds, ferric reducing antioxidant power (FRAP) methods and 1,1-diphenylpicrylhydrazyl (DPPH) radical scavenging methods were utilized. The samples were tested at varying concentrations (250–1,000 μg/mL).

##### 2.4.1.1 FRAP assay

The FRAP method (Benzie and Strain, 1996) was employed to examine the reduction capacity of the compounds. To prepare the FRAP assay, a 10:1:1 solution of phosphate buffer (pH, 7.6), TPTZ, and FeCl_3_ was heated at 70°C for 10 min and then added to the compounds. The reduction potential was determined by measuring the conversion of Fe(III) to Fe(II). The amount of Fe(II) complex in the solution was quantified by the intensity of blue color at 593 nm. A higher increase in absorbance was related to the greater reduction potential of the compounds. The FRAP was determined utilizing the standard curve of ferrous sulfate with butylated hydroxytoluene (BHT) used as a reference standard.

##### 2.4.1.2 DPPH radical scavenging assay

To evaluate the antioxidant affinity, the DPPH radical scavenging procedure was conducted as detailed by Brand-Williams et al. (1995) and Aljohani et al. (2023). Here, 1 mL of the test solution was added to 0.004% methanolic solution of DPPH (4 mL) at varying concentrations (250–1,000 μg/mL). The solution was incubated in the dark for 30 min, and absorbance at 517 nm was recorded using a UV–vis spectrophotometer. BHT served as the standard here. Higher reductions in absorbance are associated with higher antioxidant activity. The following expression was used to calculate DPPH scavenging affinity %:

$$\text{DPPH scavenging affinity }\%=\left[\left(A_{C}\;-\;A_{S}\right)/A_{C}\right]\;x\;100,$$

where A_C_ and A_S_ denote the absorbance of the control and sample, respectively. A comparative analysis was conducted by determining the inhibitory concentration at 50% inhibition (IC_50_) values using the graph between the concentration and DPPH scavenging affinity %.

#### 2.4.2 Antibacterial activity

The minimum inhibitory concentration (MIC), quantified as the minimal concentration that inhibits bacterial growth post-overnight incubation, was ascertained utilizing the protocol followed by Devi and Batra (2015). Evaluations were executed on a pair of Gram-positive microorganisms, *Bacillus subtilis* and *Staphylococcus aureus*, alongside Gram-negative species, *Salmonella typhi* and *Escherichia coli*. Initially, a stock solution of the synthesized compounds was prepared in DMSO at a concentration of 100 μg/mL. This stock solution was then serially diluted to achieve concentrations of 50, 25, 12.5, 6.25, and 3.125 μg/mL. Hinton–Muller agar was prepared and uniformly spread across sterilized Petri dishes, which were subsequently incubated to achieve solidification. Thereafter, bacterial strains were inoculated on these plates, and aliquots spanning concentrations of 3.125–100 μg/mL were administered via 5-mm paper disks. The MIC values were recorded after an overnight incubation period.

### 2.5 Molecular docking study

A molecular docking study was performed to observe the molecular interactions between the prepared compounds and the protein receptors of *S. aureus* and *S. typhi*. The cell membrane protein OmpF complex of *S. typhi* (PDB ID: 4KR4) and *S. aureus* tyrosyl–tRNA synthetase (PDB ID: 1JIL) was used as the protein for the study (Sanner, 1999; De et al., 2020). The 3D structure of the protein in PDB file format was obtained from the PDB database (https://www.rcsb.org), and the ligand molecule in PDB file format was obtained from PubChem (https://pubchem.ncbi.nlm.nih.gov/). The grid and docking parameter files (GPFs and DPFs, respectively) were prepared by using AutoDock tools. The GPF defines the size and location of the grid that will be used to search for potential binding sites on the protein, while the DPF specifies the parameters for the genetic algorithm that will be used to dock the ligand to the protein. AutoGrid is used to generate a grid map of the protein based on the GPF. Blind docking was conducted to study the protein–ligand interactions. For each docking simulation, a grid box of dimensions 126 × 126 × 126 Å was used to encompass the entire protein structure, with the center coordinates set differently for each molecule and given in Table 3. The DPF was generated using the Lamarckian genetic algorithm (LGA), with a maximum of 2,500,000 energy evaluations. All other values were used as default. This will help identify the most favorable binding sites for the ligand. The docking simulation was performed using the AutoDock genetic algorithm. The docking results were analyzed using AutoDock 4.2 and BIOVIA Discovery Studio.

## 3 Results and discussion

Various methods such as magnetic, analytical, and spectral methods were used to characterize the synthesized compounds. Table 1 summarizes the yields, colors, magnetic moments, melting points, molar conductivity, and elemental compositions of the ligand and complexes. The molar conductivity of all compounds was low, indicating that they were non-electrolytic (Geary, 1971). The ligand precursor and ligand were soluble in CH_3_OH and DMSO, while the metal complexes were soluble in DMSO.

**TABLE 1:** Physical and analytical data of synthesized compounds.

Compound	Color	Yield (%)	Molecular weight (g mol^-1^)	Magnetic moment (BM)	Molar conductivity (mho cm^2^ mol^-1^)	λ_max_ (nm)	MP	% elemental analysis calculated (found)			
							(°C)	C	H	N	Metal
**Ligand precursor**	White	84	155	–	10.1	238	53	54.04 (54.66)	3.89 (3.85)	9 (9.12)	-
**Schiff base ligand**	Light yellow	84	335	–	9.54	288	94	57.33 (57.45)	4.81 (4.92)	16.71 (16.56)	-
**Mn(II) complex**	Light pink	78	845	5.83	12.84	372	281 (decomposition temperature)	51.26 (51.56)	4.54 (4.48)	13.28 (13.56)	6.51 (6.32)
**Zn(II) complex**	Yellow	75	856	–	9.67	283	277 (decomposition temperature)	50.63 (50.72)	4.49 (4.52)	13.12 (12.98)	7.66 (7.86)

### 3.1 Characterization

#### 3.1.1 Ultraviolet–visible spectroscopy and magnetic properties

**FIGURE 1:**
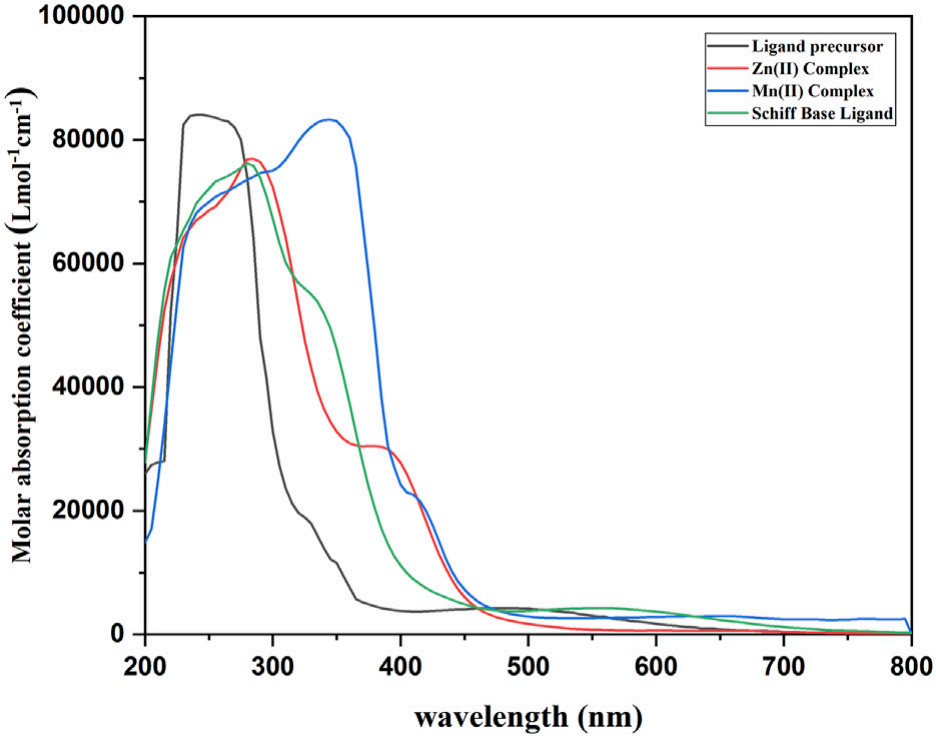
UV–vis spectra of the prepared compounds.

The ligand precursor exhibited a λ_max_ at 238 nm, likely associated with a π–π* transition. Additionally, a band at 484 nm can be ascribed to an n–π* transition, indicative of bonding with a system possessing a lone pair of electrons. In the case of the ligand, λ_max_ appears at 288 nm (π–π* transition), and the other band appears at 356 nm (n–π* transition). The emergence of a distinct shoulder band in all complexes signifies the occurrence of charge transfer bands, providing conclusive evidence for the formation of a complex between the ligand and metal ions (Damiche and Chafaa, 2017). The Mn(II) complex was found to be paramagnetic, having a magnetic moment of 5.83 BM, suggesting the octahedral nature of the complex. The Mn(II) complex exhibited λ_max_ at 372 nm and an LMCT band at 413 nm. Owing to the d-d transition bands being spin-forbidden and Laporte-forbidden, they are either absent or possess such minimal intensity that they are not observed. The same types of results were also reported by Devi et al. (2012) and Keypour et al. (2013). The Zn(II) complex was diamagnetic, and no d-d transition was observed for the complex. λ_max_ and a charge transfer band were observed at 283 and 387 nm, respectively (Figure 1). The maximum molar absorption coefficients of the ligand precursor, Schiff base ligand, Mn(II) complex, and Zn(II) complex were 84,267, 75,976, 83,560, and 75,977 Lmol^−1^cm^−1^, respectively.

#### 3.1.2 Infrared spectroscopy

FTIR spectroscopy serves as a pivotal tool for elucidating the structural intricacies of Schiff base ligands and their complexes. The infrared (IR) spectral analysis of the ligand precursor unveiled characteristic absorptions, notably the C=N stretching vibration at 1,621 cm^−1^, indicative of imine formation, and the N-H stretching at 3,515 cm^−1^, associated with amine functionalities. Notably, the C=O stretch, initially present at 1,715 cm^−1^, vanishes upon Schiff base formation, affirming the condensation of the carbonyl group with a primary amine to yield the imine linkage. Upon metal coordination, these bands undergo a discernible shift to lower wavenumbers, a phenomenon attributed to bond length augmentation. Concurrently, a new band emerges in the vicinity of 1,711–1,735 cm^−1^, ascribed to the carbonyl stretch within the acetate moiety, a common feature observed in metal–acetate complexes (Supplementary Figure S1).

Further scrutiny of the metal complexes through FT-IR spectroscopy reveals the presence of metal–ligand vibrational modes in the far-infrared region. For the Mn(II) complex, the emergence of bands at 468 cm^−1^ and 378 cm^−1^ corresponds to the Mn-N and Mn-O stretches, respectively. Similarly, the Zn(II) complex exhibits bands at 396 cm^−1^ for Zn-O and 456 cm^−1^ for Zn-N vibrations (Table 2). These bands are quintessential indicators of metal–nitrogen and metal–oxygen coordination, providing empirical evidence for the successful synthesis of the respective metal complexes.

**TABLE 2:** FTIR stretching frequencies (cm^-1^) of synthesized compounds.

Compound	ʋ(N-H)	ʋ(C=O)	ʋ(C=N)	ʋ(COO)_asymm_	ʋ(COO)_symm_	ʋ(M-N)	ʋ(M-O)
Ligand precursor	3,515	1,715	–	–	–	–	–
Schiff base ligand	3,536	–	1,621	–	–	–	–
Mn(II) complex	3,547	1,713	1,675	1,521	1,325	468	378
Zn(II) complex	3,568	1,711	1,625	1,508	1,334	456	396

The carboxylate group (from acetate) in the complexes shows two stretching bands: asymmetrical and symmetrical. The asymmetric stretching band typically appears at a higher wavenumber than the symmetric stretching band due to differences in bond strengths and the molecular vibrations associated with these modes. For the Mn(II) complex, the asymmetric stretching band is observed at 1,521 cm^−1^, while the symmetric stretching band is observed at 1,325 cm^−1^ (Abdallah et al., 2010). In contrast, for the Zn(II) complex, the asymmetric stretching band appears at 1,508 cm^−1^, and the symmetric stretching band appears at 1,334 cm^−1^ (Soliman and Mohamed, 2004); the M-O band is present in both complexes, suggesting the acetate coordination mode. The difference between the asymmetric and symmetric frequency Λ[ʋ_asymm_ (COO)- ʋ_symm_ (COO)] is large (175–200 cm^−1^), which suggests monodentate legation (Belal et al., 2015).

#### 3.1.3 ^1^H NMR spectroscopy

**FIGURE 2:**
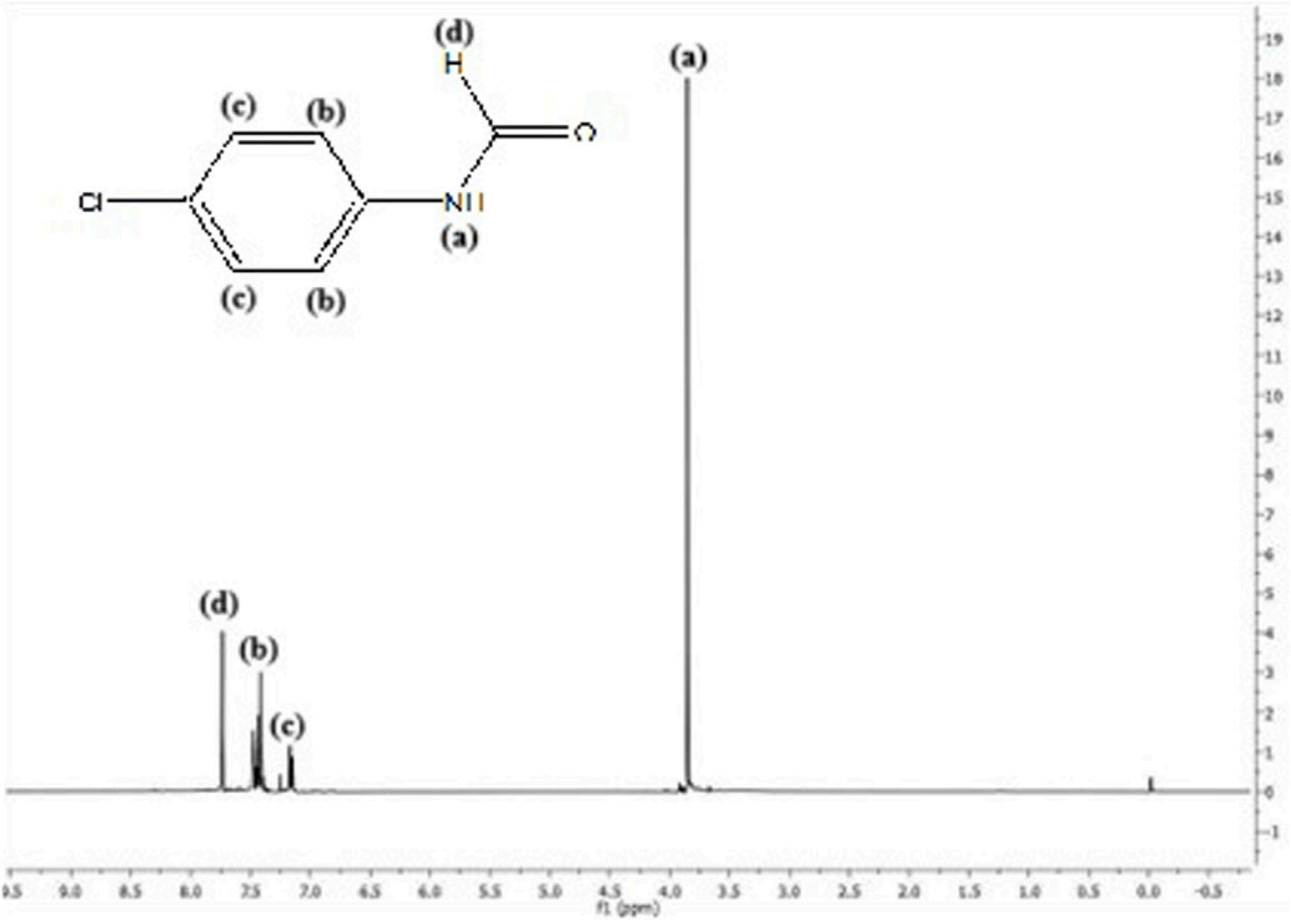
^1^H NMR spectra of the Schiff base ligand precursor where **(a)**, **(b)**, **(c)** and **(d)** represent -NH, -CH, -CH and aldehydic H respectively.

^1^H NMR spectra of the prepared compounds confirm the proposed structure (Scheme 3). For the ligand precursor, the -NH bond appears at 3.8 ppm, the aldehydic proton appears at 7.8 ppm, and signals from 7.1 ppm to 7.4 ppm represent the aromatic region (Figure 2). In the case of the Schiff base ligand, the existing band of -NH shifted toward lower chemical shift, and a new band of -CH_2_ (ethylene diamine) appeared at 1.5 ppm (Supplementary Figure S2). The Mn(II) complex did not show the NMR signals, which may be attributed to the paramagnetism of the Mn(II) complex. For the Zn(II) complex, the band due to -NH shifted toward lower chemical shifts due to chelation of the ligand to the metal, and a new band appeared at approximately 2.4 ppm due to the -OCOCH_3_ group. NMR of the ligand precursor and Zn(II) complex is given in Supplementary Material (Supplementary Figure S3).

#### 3.1.4 Powder XRD study

**FIGURE 3:**
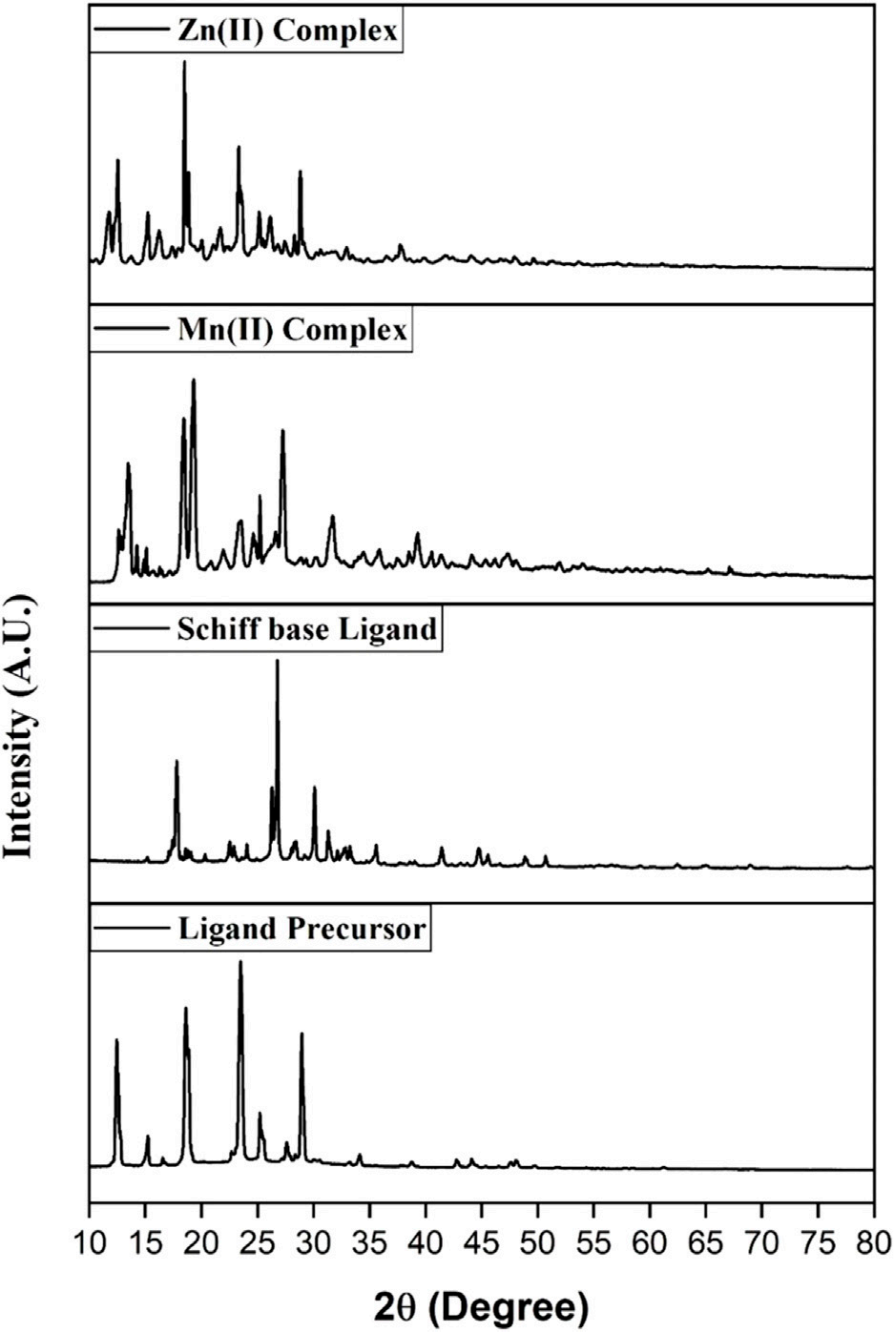
PXRD spectra of the prepared compounds.

Powder X-ray diffraction was performed in the 10 < 2θ < 80 range to study their lattice dynamics (Figure 3). The particle size (D) was obtained using the Debye–Scherrer expression:

D=Kλ/βCosθ,

where K is a constant of the Cu grid (0.94), λ denotes the wavelength of the X-ray, θ denotes the Bragg diffraction angle, and β is the integral peak width (Sundararajan et al., 2014).

The sizes of the ligand precursor, ligand, and Mn(II) and Zn(II) metal complexes were identified as nanocrystalline with 41.07, 96.6, 40.21, and 122.20 nm, respectively. The degree of crystallinity for the ligand precursor, Schiff base ligand, and Mn(II) and Zn(II) metal complexes was found to be 46.07, 30.09, 35.10, and 21.07%, respectively.

The XRD pattern reveals distinct crystalline patterns for both the ligand and its complexes, exhibiting different degrees of crystallinity. The complexes exhibit extra peaks compared to the ligand, confirming metal ion chelation and signaling complex formation (El-Megharbel and Hamza, 2022).

### 3.2 Biological activities

#### 3.2.1 Antioxidant activities

##### 3.2.1.1 FRAP assay

**FIGURE 4:**
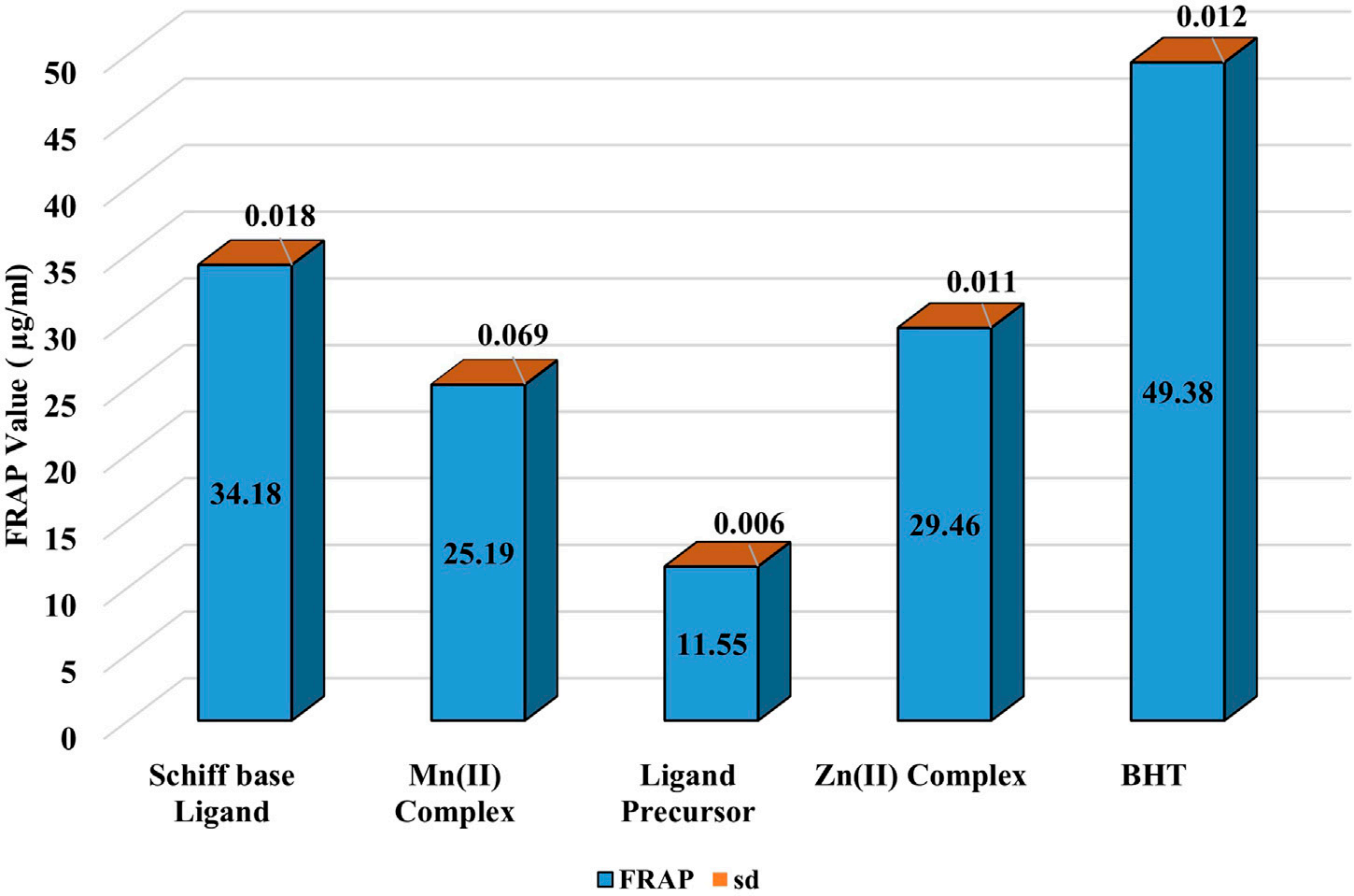
Demonstration of FRAP of the prepared compounds compared to BHT.

The ability to reduce ferric ions was evaluated through the FRAP assay. The standard curve of FeSO_4_.7H_2_O was utilized for obtaining FRAP values (Supplementary Figure S4). The findings indicated that all tested compounds possessed ferric-reducing antioxidant potential, with the ligand showing the highest reducing potential among all the compounds. The Zn(II) complex showed the highest FRAP among the metal complexes, and the activity follows the order BHT > Schiff base ligand > Zn(II) complex > Mn(II) complex > ligand precursor (Figure 4).

##### 3.2.1.2 DPPH radical scavenging activity

**FIGURE 5:**
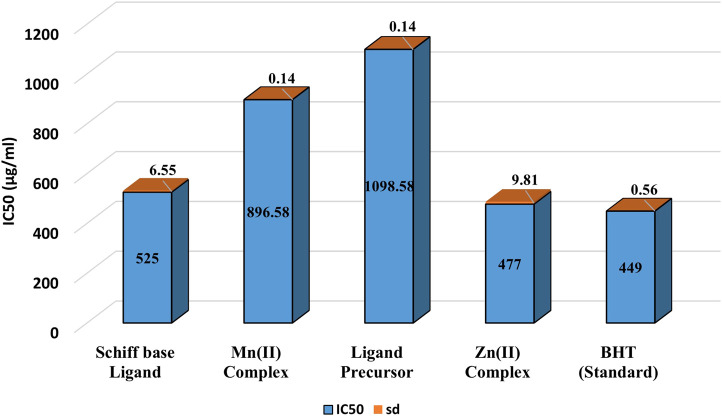
IC_50_ values of DPPH radical scavenging activity of Ligand precursor, Schiff base Ligand, Mn(II) Complex, Zn(II) Complex and Standard (BHT).

Figure 5 demonstrates the DPPH radical scavenging potential of the examined compounds. Antioxidant activity was determined by evaluating the hydrogen-donating capacity or radical scavenging potential against the stable radical DPPH. Upon hydrogen radical abstraction, a color transition from dark purple to pale yellow was noted in the DPPH solution, and this change was quantified by assessing the reduction in the absorbance at 517 nm. The results indicated that the Zn(II) complex exhibited the highest scavenging potential among the examined compounds. The general order of DPPH radical scavenging potential was as follows: BHT > Zn(II) complex > ligand > Mn(II) complex > ligand precursor.

#### 3.2.2 Antibacterial activity

**FIGURE 6:**
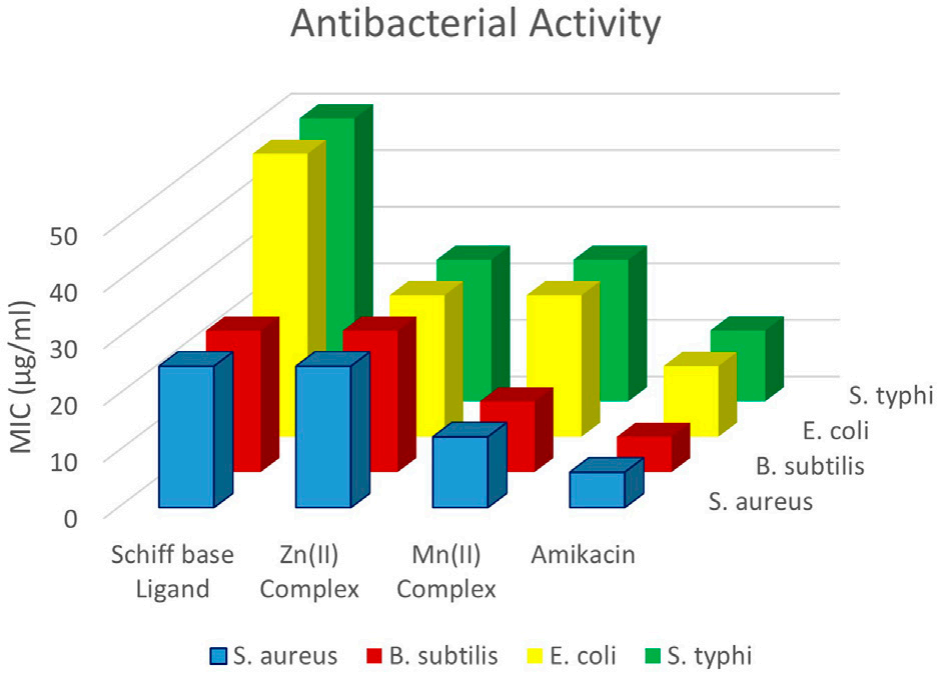
Antibacterial activity against Gram-negative bacteria (*S. aureus* and *B. subtilis*) and Gram-positive bacteria (*E. coli* and *S. typhi*).

Figure 6 illustrates the antibacterial screening results of all the tested compounds. Good antibacterial activity was observed for all the tested compounds. The metal complexes showed higher antibacterial activity than the ligand precursor and ligand. This could be attributed to Tweedy’s chelation theory, which proposes that chelation increases the bactericidal power of metal complexes as the metals share their positive charge with ligands, leading to p-electron delocalization throughout the compound. Thus, the complexes acquired a lipophilic nature, making it easier for them to permeate through membranes (Tweedy, 1964; Abu-Dief et al., 2019). Both Gram-positive and Gram-negative bacteria were tested, and the results showed that the tested compounds were more active for Gram-positive bacteria. Gram-positive bacteria are often considered to have a somewhat more permeable cell membrane because of the absence of an outer membrane, which simplifies their structure, allowing for potentially easier diffusion of molecules (Malanovic and Lohner, 2016). The highest activity was observed for *S. typhi*, although the activity of the examined compounds was lower than that of amikacin (standard). Among the complexes, the Mn(II) complex showed higher activities than the Zn(II) complex.

### 3.3 Molecular docking study

**FIGURE 7:**
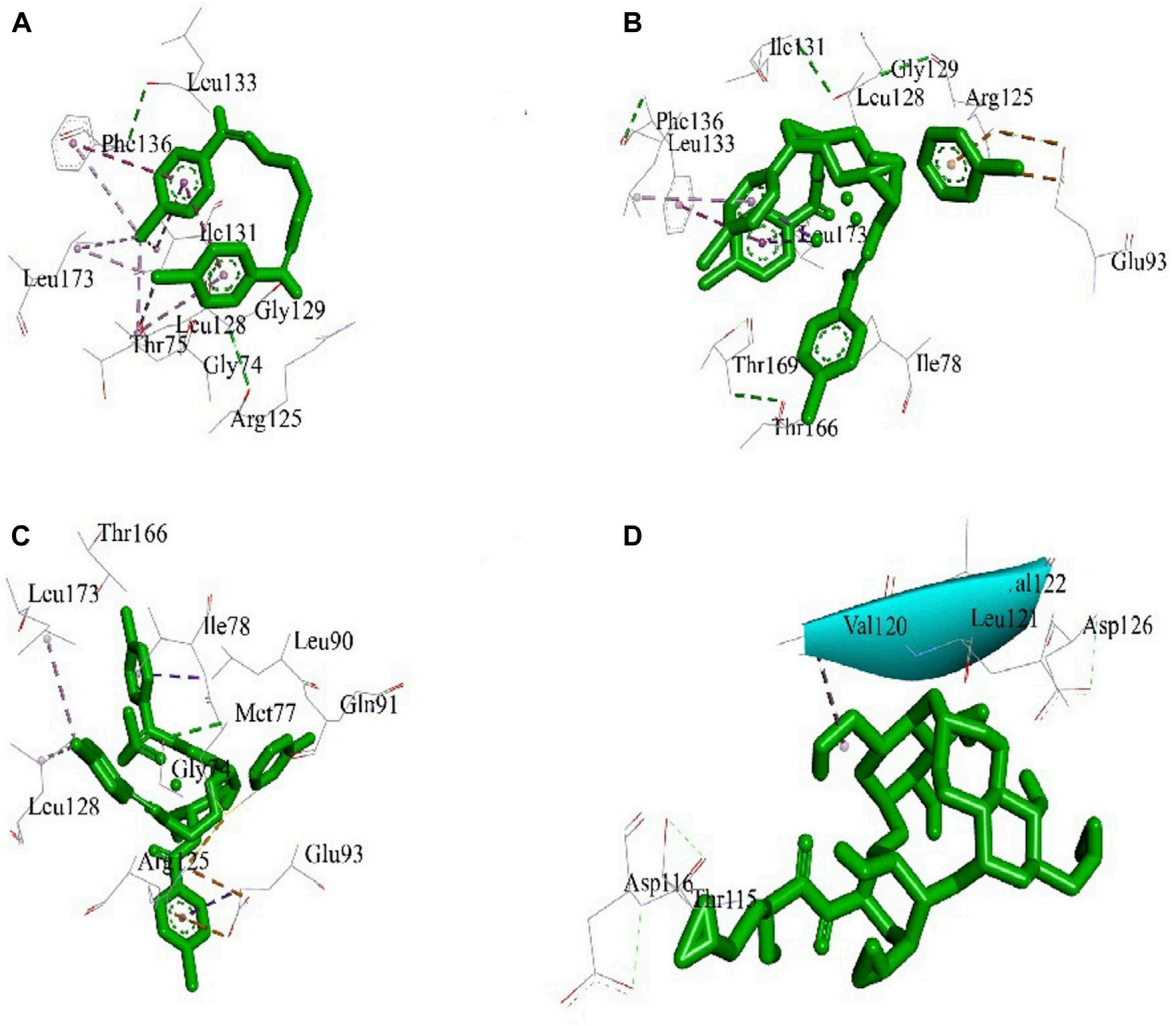
Docking interaction diagrams of the **(A)** Schiff base ligand, **(B)** manganese(II) complex, **(C)** zinc(II) complex, and **(D)** amikacin against *S. aureus* tyrosyl–tRNA synthetase.

**FIGURE 8:**
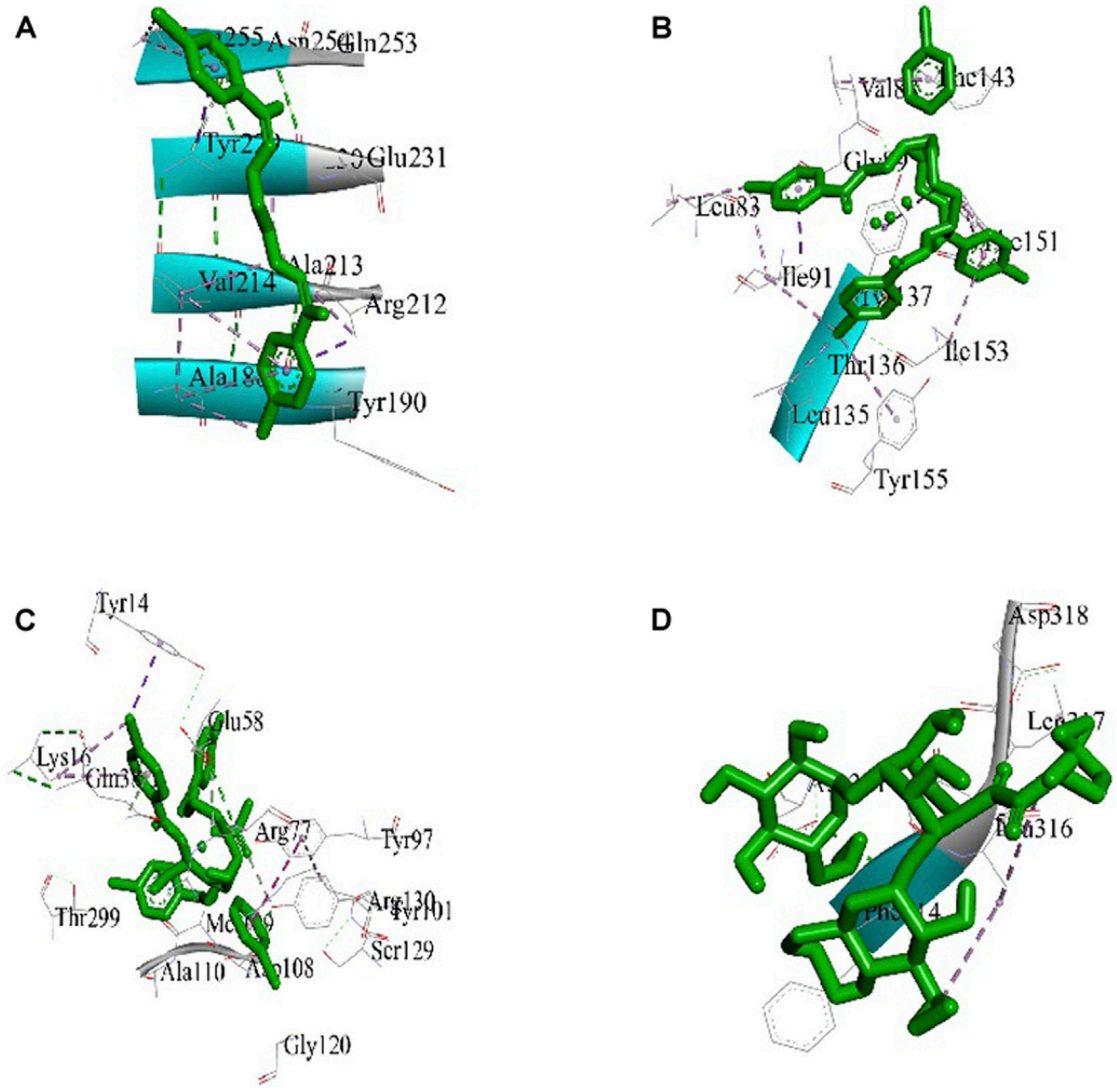
Docking interaction diagrams of the **(A)** Schiff base ligand, **(B)** manganese(II) complex, **(C)** zinc(II) complex, and **(D)** amikacin against the *S. typhi* protein OmpF complex.

The best 3D binding interaction of test compounds with the *S. aureus* tyrosyl–tRNA synthetase (Figure 7) and *S. typhi* cell membrane protein OmpF complex (Figure 8) is demonstrated. The binding energy represents the thermodynamic stability of the ligand–receptor complex. In molecular docking studies, a lower (more negative) binding energy is generally considered indicative of a more favorable and stronger interaction between a ligand (docked compound) and its target receptor (Wolohan and Reichert, 2004). These observations indicate substantial binding between the ligand and its complexes with the protein receptor, resulting in a favorable free binding energy.

In the case of the *S. typhi* protein OmpF complex, the best activity was shown by the Mn(II) complex (binding energy, −7.34 kcal/mol), followed by the Zn(II) complex (binding energy, −4.97 kcal/mol) and the ligand (binding energy, −4.47 kcal/mol). However, in the case of *S. aureus* tyrosyl–tRNA synthetase, the Mn (II)complex (binding energy, −4.43 kcal/mol) showed the highest activity, followed by the ligand (binding energy, −3.68 kcal/mol) and Zn(II) complex (binding energy, −3.28 kcal/mol) (Table 3).

**TABLE 3:** Molecular docking results of docked compounds against the *S. typhi* cell membrane protein OmpF complex and *S. aureus* tyrosyl–tRNA synthetase.

Compound (ligand)	Target receptor	Binding energy (ΛG)	Inhibition constant (ki) µM	Interactive amino acids	Grid point spacing (Å)	Center coordinate			Size coordinate		
						x	y	z	x	y	z
Schiff base ligand	*S. typhi* cell membrane protein OmpF complex	−4.47	528.16	Glu231, Ala188, Ala189, Ala213, Ala230, Tyr190, Tyr229, Leu255, Gln253, Asn254, Val214, and Arg212	0.558	−24.247	12.382	−8.438	126	126	126
Mn(II) complex	*S. typhi* cell membrane protein OmpF complex	−7.34	4.14	Tyr137, Tyr155, Thr136, Leu135, Ile91, Ile153, Leu83, Val88, Gly89, Phe151, and Phe143	0.536	−22.824	10.357	−8.438	126	126	126
Zn(II) complex	*S. typhi* cell membrane protein OmpF complex	−4.97	226.06	Ser129, Gly120, Arg77, Arg130, Asp108, Tyr14, Tyr97, Tyr101, Ala110, Gln38, Thr299, Met109, Glu58, and Lys16	0.547	−21.827	10.834	−8.438	126	126	126
Amikacin	*S. typhi* cell membrane protein OmpF complex	−5.56	84.15	Asp318, Leu317, Asp331 Leu316, Asn315, and Phe314	0.553	−24.158	9.112	−8.438	126	126	126
Schiff base ligand	*S. aureus* tyrosyl–tRNA synthetase	−3.68	2.01	Gly74, Gly129, Leu128, Leu133, Leu173, Phe136, Arg125, Ile131, and Thr75	1.000	34.913	6.348	54.727	126	126	126
Mn(II) complex	*S. aureus* tyrosyl–tRNA synthetase	−4.43	565.39	Glu93, Arg125, Thr166, Thr169, Leu128, Leu133, Leu173, Ile78, Ile131, Gly129, and Phe136	1.000	34.913	1.013	55.424	126	126	126
Zn(II) complex	*S. aureus* tyrosyl–tRNA synthetase	−3.28	3.93	Glu93, Arg125, Leu90, Leu128, Leu173, Thr166, Ile78, Gln91, Gly74, and Met77	1.000	34.913	11.323	55.742	126	126	126
Amikacin	*S. aureus* tyrosyl–tRNA synthetase	−4.36	3.41	Asp116, Glu184, Lys30, Asp97, Thr115, Gly23. Glu101, Glu184, Asp20, Glu93, Glu94, and Asp126	1.000	32.577	11.102	56.671	126	126	126

## 4 Conclusion

In summary, Mn(II) and Zn(II) complexes of the formamide Schiff base ligand, derived from ethylene diamine and 4-chloro (N-phenyl)formamide, were synthesized and characterized by various analytical, spectral, and magnetic methods. The antioxidant potential of the prepared compounds was evaluated through DPPH radical scavenging activity assay and the FRAP method, and the following general trend was found: BHT > ligand ∼ Zn(II) complex > Mn(II) complex > ligand precursor. Afterward, all the synthesized compounds underwent assessment for their antibacterial efficacy against two Gram-negative bacteria (*S. aureus* and *B. subtilis*) and two Gram-positive bacteria (*E. coli* and *S. typhi*). The Mn(II) complex exhibited better bactericidal capacity than other compounds, which was validated by molecular docking interaction study. In studies involving the *S. typhi* cell membrane protein OmpF complex and *S. aureus* tyrosyl–tRNA synthetase, the Mn(II) complex demonstrated the highest binding affinity, followed by the ligand and Zn(II) complex.

## Data Availability

The original contributions presented in the study are included in the article/Supplementary Material; further inquiries can be directed to the corresponding authors.

## Abbreviations

AAS, atomic absorption spectra; *B. subtilis*, *Bacillus subtilis*; BHT, butylated hydroxytoluene; DMSO, dimethyl sulfoxide; DPPH, 1,1-diphenylpicrylhydrazyl; *E. coli*, *Escherichia coli*; FTIR, Fourier-transform infrared; MHz, megahertz; NMR, nuclear magnetic resonance; PDB, Protein Data Bank; ppm, parts per million; PXRD, powder X-ray diffractometer; *S. aureus*, *Staphylococcus aureus*; *S. typhi*, *Salmonella typhi*; TLC*,* thin-layer chromatography; TMS, tetra methyl silane; TPTZ, 2,4, 6-tri (2-pyridyl)-*s*-triazine; UV–vis, ultraviolet–visible.
