# Supplementary figures and images for: Manganese(II) and Zinc(II) metal complexes of novel bidentate formamide-based Schiff base ligand: synthesis, structural characterization, antioxidant, antibacterial, and in-silico molecular docking study
Source: Front Chem. 2024 Jul 19;12:1414646. doi: 10.3389/fchem.2024.1414646 (PMC11294232; doi:10.3389/fchem.2024.1414646)

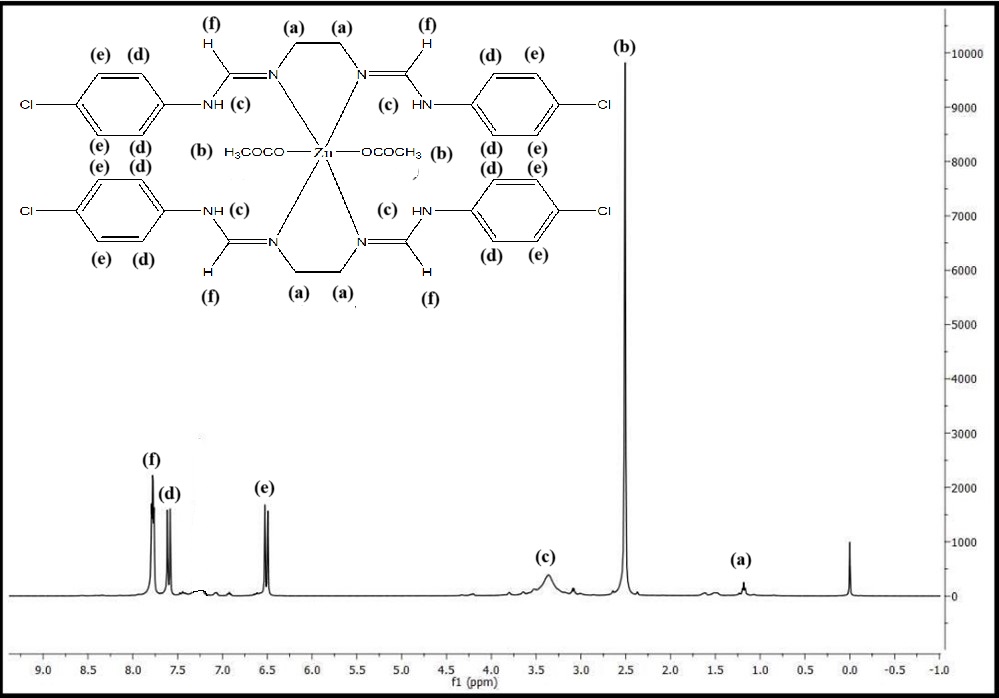

Supplement: Supplementary file 1 [file Image3.JPEG]

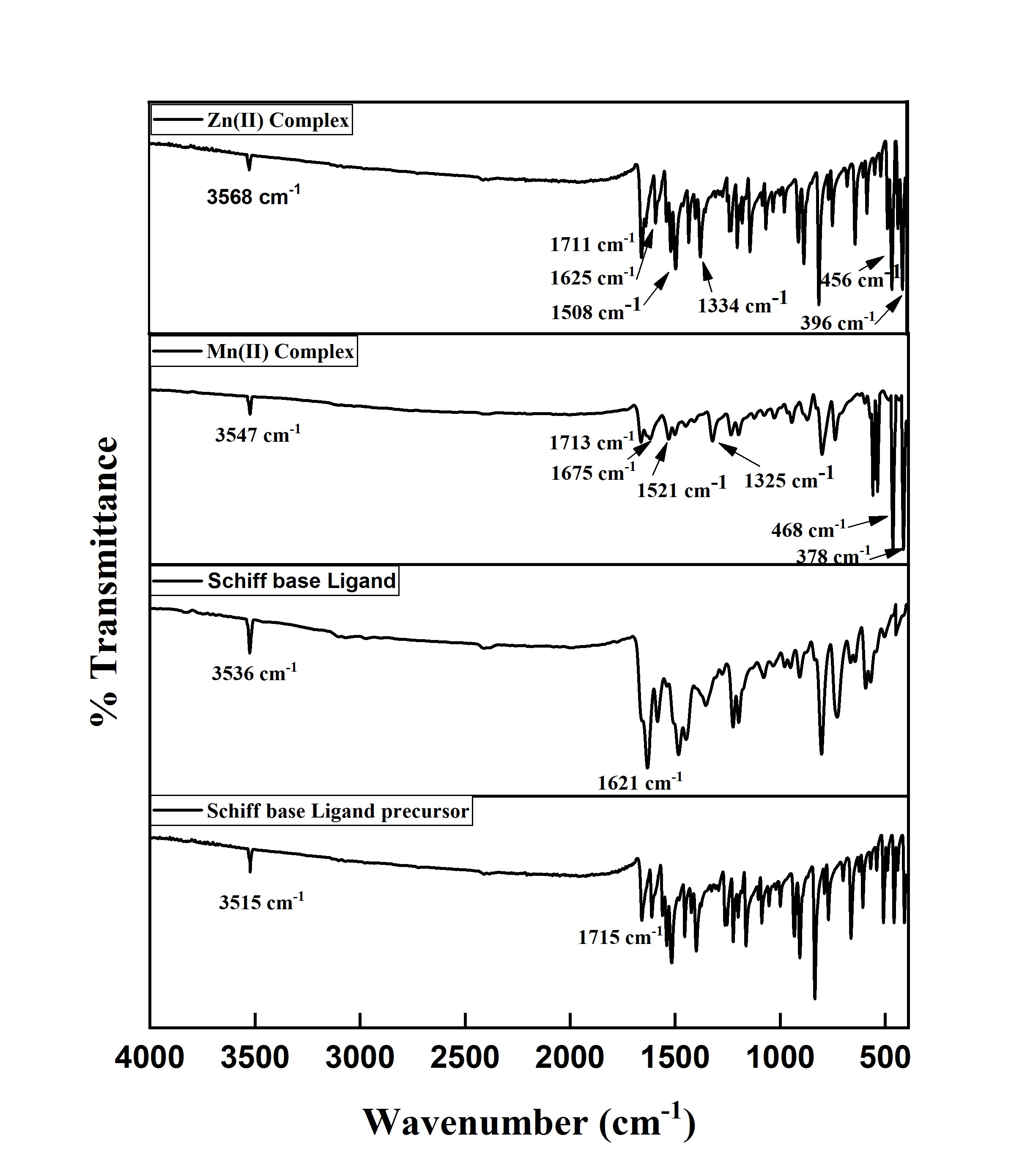

Supplement: Supplementary file 2 [file Image1.JPEG]

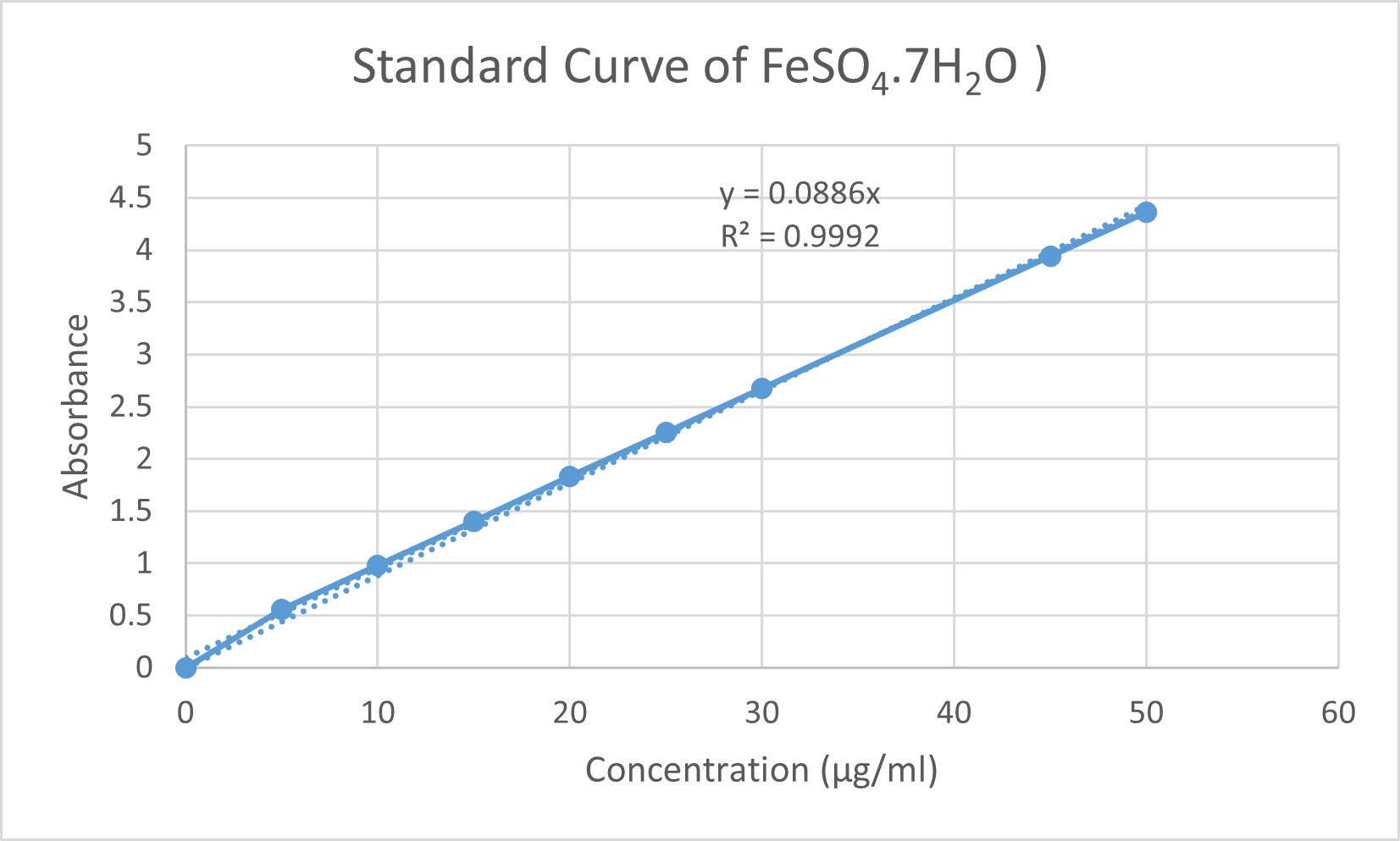

Supplement: Supplementary file 3 [file Image4.JPEG]

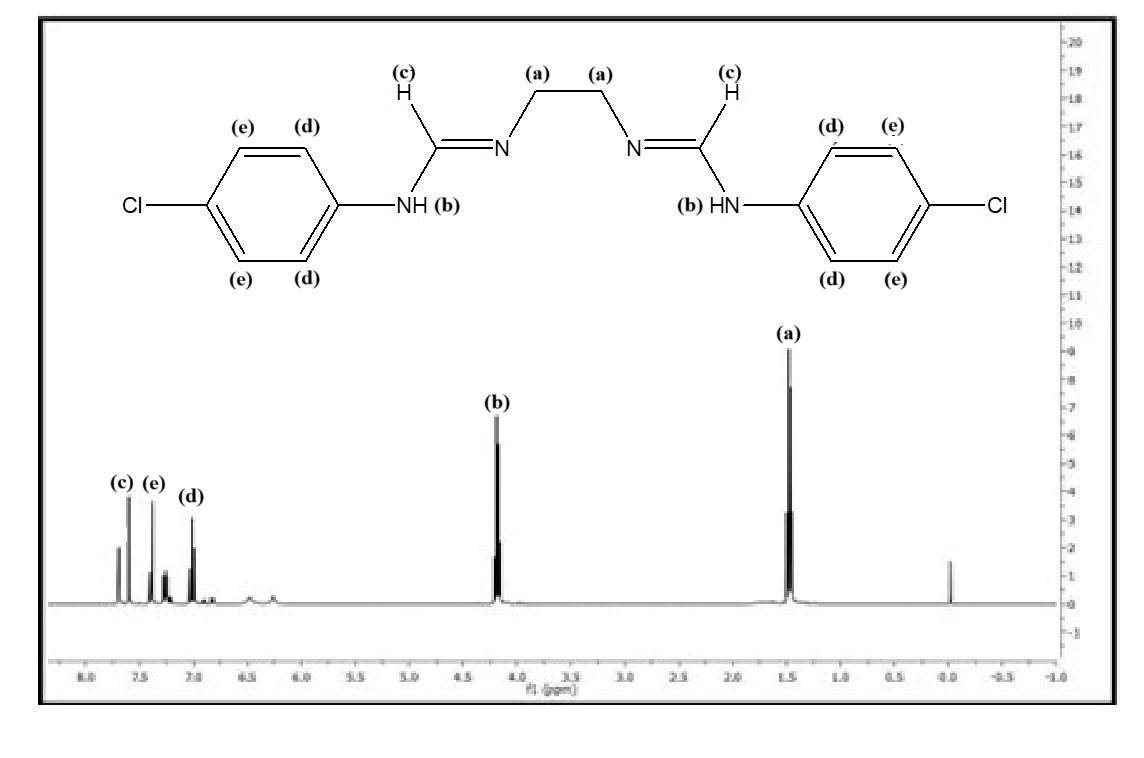

Supplement: Supplementary file 4 [file Image2.JPEG]
